# Living Emotions, Avoiding Emotions: Behavioral Investigation of the Regulation of Socially Driven Emotions

**DOI:** 10.3389/fpsyg.2012.00616

**Published:** 2013-01-21

**Authors:** Alessandro Grecucci, Cinzia Giorgetta, Nicolao Bonini, Alan G. Sanfey

**Affiliations:** ^1^Department of Psychology and Cognitive Science, University of TrentoTrento, Italy; ^2^Institute of Cognitive Science and Technology, CNR – Consiglio Nazionale delle RicercheTrento, Italy; ^3^Department of Economics and Management, University of TrentoTrento, Italy; ^4^Behavioural Science Institute, Radboud University NijmegenNijmegen, Netherlands; ^5^Donders Institute for Brain, Cognition and Behavior, Radboud University NijmegenNijmegen, Netherlands

**Keywords:** dictator game, emotion regulation, mentalizing

## Abstract

Emotion regulation is important for psychological well-being. Although it is known that alternative regulation strategies may have different emotional consequences, the effectiveness of such strategies for socially driven emotions remains unclear. In this study we investigated the efficacy of different forms of reappraisal on responses to the selfish and altruistic behavior of others in the Dictator Game. In Experiment 1, subjects *mentalized* the intentions of the other player in one condition, and *took distance* from the situation in the other. Emotion ratings were recorded after each offer. Compared with a baseline condition, *mentalizing* led subjects to experience their emotions more positively when receiving both selfish and altruistic proposals, whereas *distancing* decreased the valence when receiving altruistic offers, but did not affect the perception of selfish behavior. In Experiment 2, subjects played with both computer and human partners while reappraising the meaning of the player’s intentions (with a human partner) or the meaning of the situation (with a computer partner). Results showed that both contexts were effectively modulated by reappraisal, however a stronger effect was observed when the donor was a human partner, as compared to a computer partner. Taken together, these results demonstrate that socially driven emotions can be successfully modulated by reappraisal strategies that focus on the reinterpretation of others’ intentions.

## Introduction

Recent experimental evidence suggests that emotion regulation strategies play a key role in helping individuals to adapt to and master social interactions (Gross, 2002; Ochsner et al., 2002; Gross and John, 2003). Indeed, our ability to regulate emotions when interacting with others is considered to be a crucial dimension of both emotional intelligence (Mayer and Salovey, 1997; Lopes et al., 2011), and good mental health (Gross, 2002). Broadly speaking, emotion regulation refers to a set of processes by which “individuals influence which emotions they have, when they have them, and how they experience and express these emotions” (cf. Gross, 1999). Previous studies have examined the processes that individuals use to influence the emotions they generate, when they do so, and how these emotions are experienced or expressed (Gross, 1998). Despite the extensive literature on emotion “self regulation,” which focuses primarily on the regulation of basic emotions such as fear and disgust in relation to visual stimuli (see Ochsner and Gross, 2005), evidence of emotion regulation in social interactive situations is still poorly understood. An important experimental question is whether emotion regulation can be applied to social interactive contexts, and in particular whether the same regulatory strategies that are useful in self regulation can also be applied in interpersonal situations. This information may provide deeper understanding of psychiatric disorders characterized by serious disturbances in social functioning such as borderline personality disorder (Gunderson, 2007), avoidant personality disorder (Leising et al., 2006), or schizotypic spectrum disorders (Ballon et al., 2007).

These socially driven emotions have been recently explored by asking about the emotional regulation of subjects when looking at pictures depicting social scenes (Koenigsberg et al., 2011; Vrtickaa et al., 2011). While the methodology was similar to the “standard” studies, here the researchers employed a subset of International Affective Pictures depicting scenes with social features (e.g., people in situations of abuse, aggression…) rather than general emotional pictures. Participants were asked to reappraise emotions elicited by these social scenarios, but importantly they were not exposed to the actual emotions which stem from real social situations. Studying the regulation of actual social situations is particularly important given the failure to regulate interpersonal responses often seen in clinical disorders (Phillips et al., 2003; Ochsner and Gross, 2008).

To study real interactive situations, one popular approach has been to examine emotion regulation strategies applied to tasks derived from Game Theory. Game theory explores situations of conflict and cooperation between decision-makers (Myerson, 1997), offers well-specified models for the investigation of social exchange (Sanfey and Dorris, 2009), and can assess how social factors such as reciprocity, equity, and bargaining can affect our emotions and subsequent decisions. Several studies have used game theoretic approaches to study emotion regulation in interactive contexts (e.g., van’tWout et al., 2010). In one other example, Grecucci et al. (2012) asked subjects to reappraise their emotions when interacting with a partner who was making fair or unfair monetary offers, utilizing the classic Ultimatum Game task (Guth et al., 1982). Here, subjects’ decisions were strongly modulated by the reappraisal strategy used, with fewer rejections of unfair offers when down-regulating emotions and increased rejections when up-regulating emotions. Using fMRI demonstrated that this affective modulation was correlated with activity in the insula, a brain region previously shown to be involved in the aversive reactions elicited by unfair offers (Sanfey et al., 2003). Specifically, the posterior part of the insula showed a similar pattern of activation as was observed behaviorally (less activity for down-regulation and more for up-regulation, as compared to a neutral baseline).

Here, we aim to extend the above study by testing how social norms (such as fairness, equality, and prosocial behavior), and in particular their violations, affect our emotional reactions in an interactive context. The Grecucci et al. (2012) study showed that emotion regulation can successfully modulate economic decision-making, but an open question is what emotions are actually being regulated? In the present study we use the Dictator Game with participants in the role of receiver in order to explore how we react emotionally to social norms, both when these norms are and are not violated. The Dictator Game (Kahneman et al., 1986) involves two players, one of whom is asked to divide up a specified sum of money (usually €10 or the equivalent). The first player (Allocator) is free to make any possible division of this amount, and the second player (Recipient) simply receives whatever is proffered by the Allocator. Importantly therefore, the emotional reactions of the Recipient take place in the absence of any decision. Theories on social preference argue that people display “inequity aversion” (Fehr and Gächter, 2002) when exposed to unfair divisions of money, as are often demonstrated in the Dictator Game when the Allocator keeps more money than he/she gives away. Even though there is no commonly agreed standard for what constitutes “fair” behavior (Cornelissen et al., 2011), people expect others to balance self-interest with prosocial tendencies, resulting in approximately fair divisions. But what if our partners violate such expectations? Do we feel disappointed in such behaviors? Do we get angry at them? And more importantly, are emotion regulation strategies effective in modulating such complex socially induced emotions? These questions will be addressed in this study.

A further issue to examine here is whether different strategies have similar effects on the regulation of socially driven emotions. Of the set of strategies studied in the experimental literature of self regulation, the most well-characterized is that of reappraisal. This strategy involves reinterpreting the meaning of a stimulus in order to change one’s emotional response to it (Gross, 1999), with subjects typically asked to build an interpretation of the emotional stimulus in such a way as to decrease their emotional response. Behavioral studies have shown that reappraisal is one of the most flexible, adaptive, and commonly employed strategies for regulating negative emotional responses (Gross, 2002). Importantly, this strategy has been linked to the maintenance of well-being (Gross and John, 2003), and a recent study from our group (Grecucci et al., 2012) showed that this strategy is also effective in modulating social decision-making (in the context of Ultimatum Game behavior). In particular, we showed that *reappraisal of the intentions of the other player*, or mentalizing-reappraisal, was effective in changing interpersonal reactions (punishment behaviors) toward unfair behaviors. Making sense of social interactions requires inferring intentions, beliefs, and desires (i.e., mentalizing; see Frith et al., 1991; Frith and Frith, 2003), and this concurs with a recent study that demonstrated mentalizing abilities at work when making value-based decisions (Evans et al., 2011). Importantly, mentalizing has an effect of regulating our emotions (Sharp et al., 2011). The question here then is if this version of reappraisal can regulate socially driven emotions in the absence of a decision. Though there are also other strategies that people often use when facing emotion-eliciting situations, not all strategies are equally effective in producing healthy emotion regulation. For example, “emotional suppression,” a strategy by which individuals suppress every expression of the ongoing emotion by limiting awareness of the emotional experience (Gross, 2002), can result in diminished control of emotion, interpersonal functioning, memory, well-being, and greater depressive symptomatology (Gross and John, 2003). Another strategy that, although perhaps effective in the short-term, may be detrimental in social-interpersonal contexts is “distancing,” whereby subjects detach themselves from feelings and behave as neutral observers. Distancing has proven to be effective in reducing self-reported simple negative emotions (Gross, 2002; Ochsner et al., 2002, 2004; Kalisch et al., 2005; Eippert et al., 2007). However, distancing may also reduce positive emotions (Beauregard et al., 2001; Kim and Hamann, 2007), leading subjects to flatten their emotional reactivity in a maladaptive way, in a similar way to schizoid or avoidant personality disorders (Leising et al., 2006). Even if there is some evidence that distancing can be an effective strategy in modulating emotions when looking at emotional pictures (Kalisch et al., 2005; Koenigsberg et al., 2011), it may not be useful or healthy when interacting directly with people. Suppression is a qualitatively different strategy, as it focuses on the “expression of emotions” (Gross, 2002), whereas both mentalizing and distancing are strategies focused on “reappraising” the events when the emotion is generated but not yet expressed. Both mentalizing and distancing can be defined as interpersonal strategies that “focus on the other,” whereas suppression is a more self-focused strategy. For these reasons we selected distancing as a control strategy for one of particular interest, mentalizing.

A final unresolved issue is whether emotion regulation acts upon valence, upon arousal, or on both, and more importantly, if arousal can be decreased or increased according to the valence of the experienced emotion. The vast majority of previous studies (Ochsner et al., 2002, 2004) have used simple emotional ratings regarding the pleasantness of the experienced emotion, without trying to separate these two relevant dimensions according to current theories of emotion (Lang and Bradley, 2010). The particular task used in the present study will permit us to explore these issues by using a continuum of offers that may elicit emotions from unpleasant to pleasant, tested for both their valence and arousal effects. We predict that if the strategy is able to increase the valence (e.g., increasing positivity), arousal will be increased as well, making subjects experience a positive emotion at its most vivid. However, in case of a decrease of valence (when negative emotion is experienced), arousal should decrease to prevent a painful experience of the emotion itself. Therefore, in the present study we will first test the notion that interpersonal emotion regulation is possible. While previous studies used only pictures of social scenes (Koenigsberg et al., 2011), emotions elicited in real social interactions may well be of a qualitatively different nature than those experienced while watching unpleasant images. We have previously explored social interactive emotions elicited by the Ultimatum Game (Grecucci et al., 2012), however the effect of emotion regulation was indirectly assessed by the effect produced on socioeconomic decisions (“regulated decisions”) and not on the emotions elicited themselves. Here, we will use the Dictator Game to elicit pleasant and unpleasant social emotions, without giving players the possibility to punish the proposers’ unfair behavior. We predict an effect of emotion regulation on both the valence and arousal of the experienced emotions as compared to a baseline condition.

Secondly, we examine whether different strategies are equally effective in promoting emotion regulation. Therefore, we will test two different emotion regulation strategies: mind-of-another-reappraisal, or *mentalizing*, and *distancing*. We predict a positive effect of the mentalizing strategy on emotional ratings for which the valence becomes less unpleasant, whereas we expect that distancing is not effective in decreasing the unpleasantness of negative emotions. An additional hypothesis is that distancing will also have an effect of flattening emotional reactivity more generally. These first two aims are tested in Experiment 1.

Thirdly, we will test whether emotion regulation when interacting with a human partner is different when interacting with a non-human partner. In both contexts the strategy is the same, applied to monetary offers from both human and computer donors respectively. We expect that interpersonal emotion regulation is superior when reappraising the emotions elicited from a human as opposed to a non-human partner due to the “mentalistic” nature of the strategy used. If this is the case, this will be further confirmation of the importance of interpersonal abilities on emotion regulation of socially driven emotions, as predicted by theory (Fonagy, 2006). This aim will be tested in Experiment 2.

Fourthly, across both experiments we will examine both arousal and valence dimensions, examining differences in how alternative emotion regulation strategies (Experiment 1) and alternative contexts (Experiment 2) can affect our emotional experience. Previous experiments did not make a clear distinction between valence and arousal effects of emotion regulation. In addition to the effect of strategy on the perceived valence we also expect an effect on arousal, as it is an important dimension of the emotional experience. In particular, we predict different effects on valence and arousal according to the specific strategy used. As mentalizing involves the reinterpretation of the event, we expect a strong change on the perceived valence, but less on arousal. On the contrary, as distancing is more focused on putting oneself in a detached perspective, here we expect a stronger effect on the arousal dimension, and less on valence, as no cognitive operation is required for the evaluation of the event.

## Experiment 1

This experiment will examine the effect of regulation on socially driven emotions by employing two strategies, those of mentalizing and distancing.

### Methods

#### Participants

Twenty-two participants (11 males) from the local area participated in the study, with a mean age of 23.95 (SD ± 1.43) years. The local ethics committee approved the study and all participants provided written informed consent after the procedures had been fully explained.

#### Dictator game

**Figure 1:**
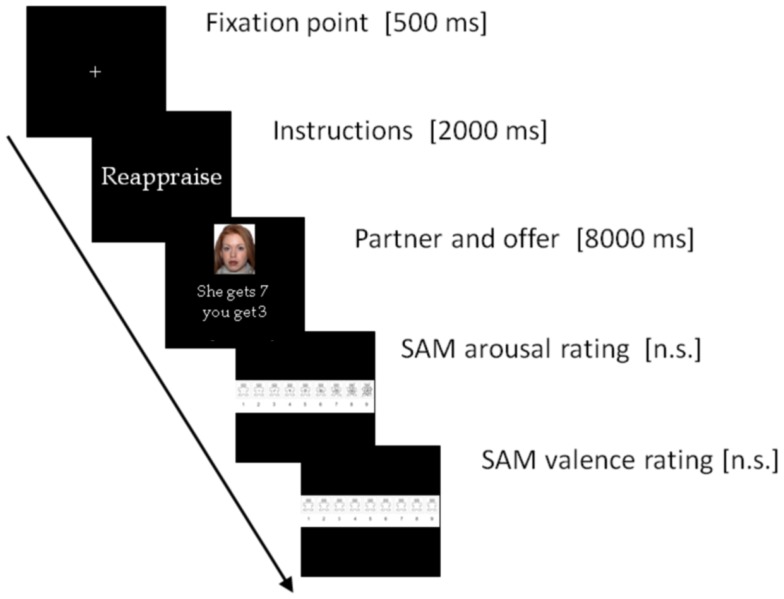
**A timeline of the events presented on each trial**. Subjects’ responses on valence and arousal ratings were recorded.

After providing informed consent, participants were first instructed as to the nature and rules of the Dictator Game. Participants were told that they would play this game as recipient with a different player in the role of the allocator on each trial. Sixty trials were presented, though participants were not informed of the total number of rounds in advance. Each round involved receiving a proposal concerning a 10€ amount. The offers included four repetitions of five possible offers (1€, 2€, 3€, 4€, and 5€ out of 10€), for a total of 20 offers for each of the three conditions (look, mentalizing, and distancing). The emotion regulation conditions were blocked and counterbalanced across participants. This was done to prevent any substantial task switching and carry over effects from one strategy to another. The offer types and pictures of Allocators were completely randomized inside each block. The task instructions emphasized that the different partners in the game would play the game independently of each other, and participants were led to believe the offers were previously recorded from real partners. Participants played the game using a computerized version of the task. The timeline of each run involved the presentation of a fixation point for 500 ms, then the instruction of the regulation strategy to be applied appeared for 2000 ms, followed by the face of the proposer and the proposal itself for 8000 ms, leaving the time to apply the strategy. After this, they were asked to rate their emotions separately on two scales (one for arousal and one for valence) using a visual analog scale known as the Self Assessment Manikin (Lang, 1994). No time constraints were given for these two events, and participants were told they would be paid a percentage of what they received during the game. See Figure 1 for a timeline.

#### Emotion regulation instructions

Before beginning the game, participants were instructed that they would have to use specific cognitive strategies upon the receipt of an offer. A written protocol describing each of the two strategies was provided. Following Gross (1998), a general reappraisal definition was given as “interpreting potentially emotion-relevant stimuli in unemotional terms,” in particular to make them less negative. An example was presented showing a picture depicting a crying woman. Participants were told that the way they interpret an event will affect the way they feel. For example, if they think that the woman is in great pain because she is mourning a loved one’s death they may feel upset, but if they think that the woman is merely tired or suffering from a headache they may feel less distressed by that event. After this example, participants were told to make an effort to reinterpret the event as less negative. They were then given the instructions of the Dictator Game and told they had to translate this strategy into the context of this game. To apply reappraisal to the Game they were asked to focus on the mind of the Allocator in order to build an interpretation of the intentions behind players’ behavior. This reinterpretation of their intentions was meant to be less negative. Some examples were given (“*he is not that stingy, probably does not have so much money to give me*,” “*this is the best he can do*” etc.). We define this kind of reappraisal focused on others’ minds as “mentalizing,” in other words an effort of generating possible explanations of the intentions of others.

For the other strategy, distancing, they were told that how involved they feel in a situation will affect their perceived distress. A picture was then presented depicting a bloody fight between police and terrorists, and they were told that if they feel themselves affected by this situation they probably will feel scared and worried, whereas if they think that that situation is far from their lives and not connected at all with them, they will feel quite neutral in relation to that event. After this, subjects were told how to apply this strategy to the context of DG. Some examples were given, such as (“*this proposal won’t affect me*,” “*I don’t care*”).

Importantly, distancing was meant to be an avoidance-based strategy, meaning that subject had to put themselves in a detached perspective, whereas mentalizing was meant to be an effort of connection with the others. Finally, for the “look” condition, they were to simply allow themselves to respond naturally, without any effort of interpretation.

Before beginning the first block of DG, we verified that participants understood the respective emotion regulation instructions by asking each to verbalize what they would do when confronted with different offers. A practice session proceeded every block.

#### Questionnaires

At the conclusion of the experiment, participants were asked to rate their emotional state on a 9-point Likert scale when they received the prototypical example of a very unfair offer (1€ out of 10€), and fair (5€ out of 10€). Moreover, effectiveness of change of emotional responses for both strategies was rated again on a 9-point Likert scale. Thinking strategies adopted during the experiment were also recorded for both strategies. This was done to ensure that participants understood the instructions and then applied them in a coherent manner according to the training instructions.

### Results

#### Emotion ratings in the dictator game

**Figure 2:**
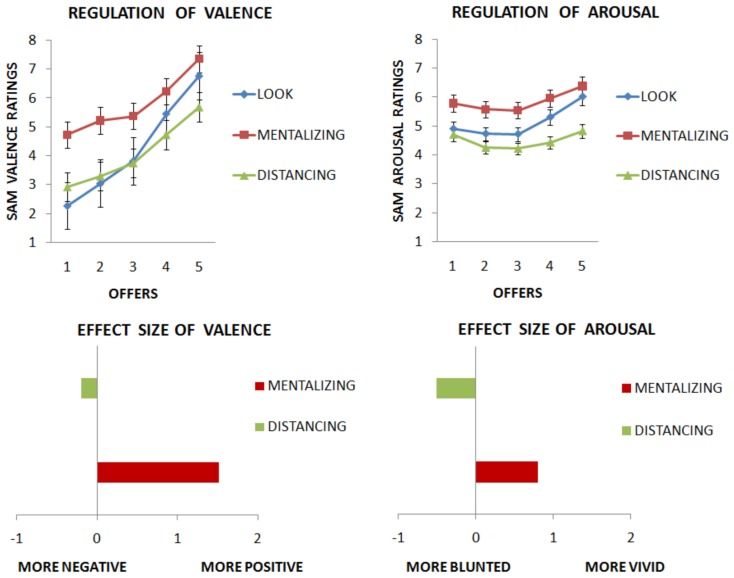
**Results from Experiment 1 are presented**. (Upper part) Valence (graph on the left) of emotions associated with both altruistic and selfish behaviors is increased when subjects mentalized the intentions of players, but not when they took the distance from them. Arousal (graph on the right) was affected by strategies. (Lower part) Results from size effects of valence and arousal are shown. See text for further information.

We first examined if the affective ratings were different across the emotion regulation and baseline conditions. We computed two separate ANOVAs, one for valence and one for arousal, each with Strategies (mentalizing vs. distancing vs. look), and Offers (1€, 2€, 3€, 4€, and 5€) as factors. Analysis on valence returned a significant main effect of Strategy [*F*(2, 42) = 41.309, *p* < 0.0001], and of Offers [*F*(4, 84) = 101.513, *p* < 0.0001], as well as a significant interaction [*F*(8, 168) = 5.817, *p* < 0.0001]. Next, Bonferroni-corrected *post hoc* tests with participants’ subjective ratings as dependent variables were computed, comparing each strategy for every offer. Three comparisons were significant for mentalizing as compared with look: for €1: (*p* < 0.05), diff: −2, 45; for €2: (*p* < 0.05), diff: −2, 17; for €3: (*p* < 0.05), diff: −1, 55; suggesting that mentalizing decreased the unpleasantness of the unfair offers (€1, €2, and €3). One comparison was significant for distancing as compared with look €5: (*p* < 0.05), diff: 1.07, suggesting that distancing decreased the valence of the most fair offer (e.g., perceived as less positive). The differences between mentalizing and distancing were all significant (all *p* < 0.05; see Figure 2).

Then, we computed ANOVA on arousal ratings. This returned a significant main effect of Strategy [*F*(2, 42) = 5.810, *p* < 0.01], and of Offer [*F*(4, 84) = 7.203, *p* < 0.0001]. However, the interaction failed to reach significance [*F*(8, 168) = 1.376, *p* = 0.21]. No further analyses were run on arousal ratings. See Figure 2.

To further explore the effect produced by each strategy, we computed the effect size of each strategy, calculated as the difference between the strategy and the baseline look condition, collapsing for all offers. In terms of valence, the mentalizing strategy returned a strong effect of 1.51 points toward more positive perception of the interaction with the partner, whereas distancing was less effective, producing a small effect of −0.19 in the direction of perceiving the events as less positive. In terms of arousal, the mentalizing produced an effect of 0.8 points in the direction of perceiving the emotions as more vivid, whereas the distancing strategy returned an effect of −0.51 points toward a more blunted perception of emotion. As expected, mentalizing had a stronger effect on valence as compared to distancing. When considering arousal, both strategies were effective in altering the ratings, however, they acted in opposite directions. Mentalizing increased arousal, whereas distancing decreased it. See Figure 2 and Table 1.

**Table 1:** **Experiment 1: results from the experiment and from the questionnaires**.

Experiment ratings			

	Regulation of valence		
	Look	Mentalizing	Distancing
€1	2.27 (1.19)	4.72 (1.75)*	2.92 (1.51)
€2	3.04 (1.29)	5.21 (1.58)*	3.29 (1.38)
€3	3.81 (1.37)	5.37 (1.60)*	3.75 (1.27)
€4	5.45 (1.77)	6.22 (1.72)	4.72 (1.49)
€5	6.76 (2.15)	7.35 (1.94)	5.68 (1.70)*
Effect size of valence		+1.51*	−0.19*

	**Regulation of arousal**		
	**Look**	**Mentalizing**	**Distancing**

€1	4.89 (2.48)	5.78 (1.98)	4.70 (2.78)
€2	4.72 (2.12)	5.57 (1.84)	4.25 (2.23)
€3	4.71 (1.89)	5.54 (1.75)	4.21 (1.99)
€4	5.30 (2.01)	5.96 (1.97)	4.43 (2.08)
€5	6.02 (2.31)	6.38 (2.11)	4.82 (2.22)
Effect size of arousal		+0.8*	−0.51*

**Questionnaires**			

**1€ Offer**		**5€ Offer**	

Anger	5.45 (2.80)	Anger	1.45 (0.91)
Disgust	5.04 (2.53)	Disgust	1.77 (1.19)
Surprise	4.54 (2.38)	Surprise	5.90 (2.18)**
Happiness	2.22 (1.65)**	Happiness	7.09 (1.63)**
Sadness	5.09 (2.58)	Sadness	1.72 (1.24)
Disappointment	6.27 (2.31)	Disappointment	1.5 (0.74)

*Indicates a significant difference.

**Indicates a significant difference from the other emotions inside every offer.

#### Questionnaires

Emotional ratings when receiving both very fair (€5) and very unfair (€1) offers were entered into an ANOVA for each of the six emotions inquired about (anger, disgust, surprise, sadness, happiness, and disappointment). Analysis returned a significant main effect of offer [*F*(2, 21) = 28.487, *p* < 0.0001], and of emotion [*F*(5, 105) = 9.071, *p* < 0.0001], as well as a significant interaction [*F*(5, 105) = 50.349, *p* < 0.0001]. Bonferroni-corrected *post hoc* tests were then computed with participants’ subjective ratings as dependent variables, comparing for each emotion and every offer. For the unfair offer, the strongest emotion elicited was disappointment (score: 6.28), followed by anger (5.45), sadness (5.09), disgust (5.04), surprise (4.54), and happiness (2.22). Disappointment, anger, disgust, sadness, and surprise differed from happiness (*p* < 0.05), though not from each other (*p* > 0.05).

For the fair offer, the strongest emotion elicited was happiness (7.09), followed by surprise (5.9) disgust (1.77), sadness (1.72), disappointment (1.5), and anger (1.45). Happiness and surprise differed from all other emotions (*p* < 0.05), but not from each other. When comparing between fairness levels, the emotions of anger, disgust, happiness, sadness, and disappointment significantly differed (*p* < 0.05), whereas surprise did not (*p* > 0.05).

**Figure 3:**
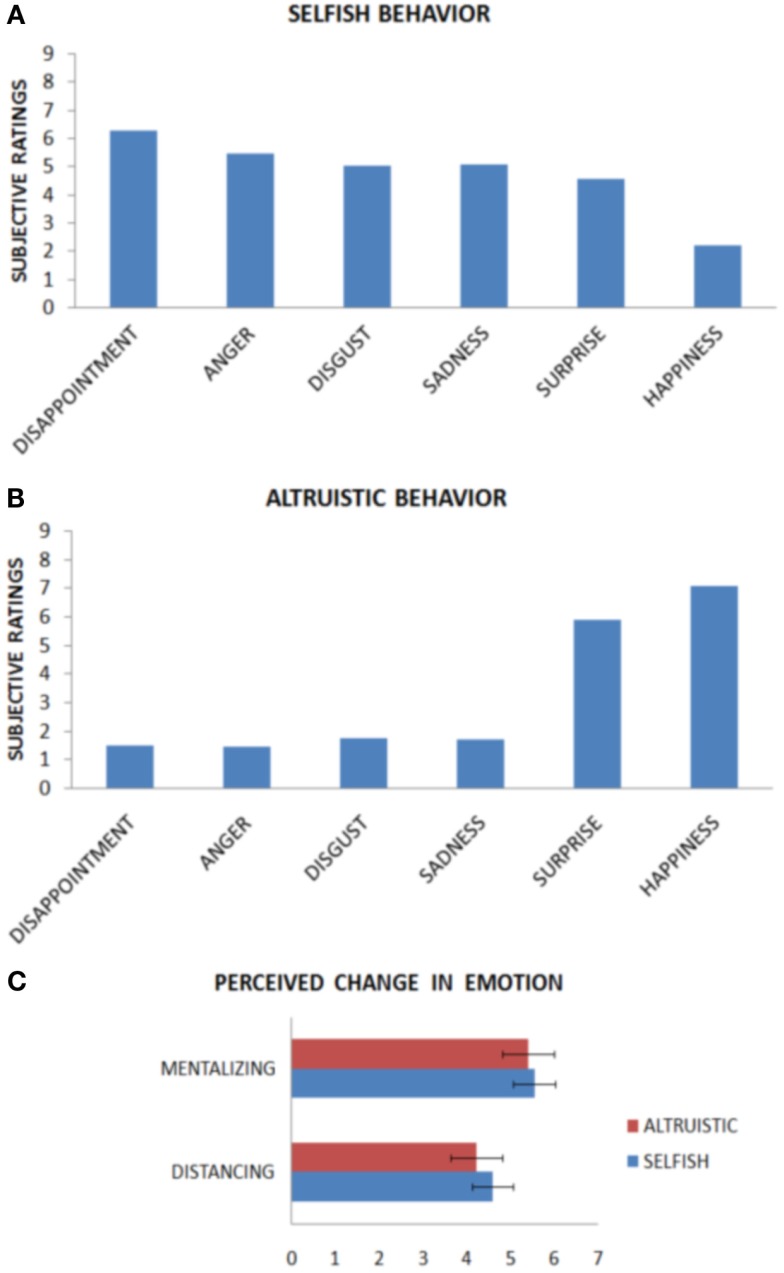
**Results from questionnaires after Experiment 1 are presented**. Subjective ratings when observing a selfish behavior **(A)** and an altruistic behavior **(B)** indicate that emotion regulation involved specific emotions. Moreover, subjects experienced large changes in their emotions when applying the strategies **(C)**, with mentalizing being superior to distancing.

We can therefore conclude that the main emotions elicited by the interpersonal context of the Dictator Game when treated unfairly was primarily disappointment, with disgust, sadness, and anger invoked to a lesser extent. These emotions may be the ones regulated during the strategy of mentalizing. We can also conclude that the main emotion elicited by fair treatment was mainly happiness, but also surprise was invoked by the altruistic behavior. See Figures 3A,B and Table 1.

After the experiment, participants were also asked to evaluate on a 9-point Likert scale how much they felt their emotions changed as a function of the two emotion regulation strategies. In the mentalizing condition they rated their emotion change with strength of 5.54 (SD ± 2.17) when confronted with selfish behavior, and 5.41 (SD ± 2.30) when confronted with altruistic behavior. In the distancing condition they felt their emotions changed with a strength of 4.59 (SD ± 2.30) when confronted with selfish behavior, and 4.22 (SD ± 2.24) when confronted with altruistic behavior. Emotional ratings when applying the two strategies to both fair (€5) and unfair (€1) offers entered an ANOVA. Analysis returned a significant main effect of strategy [*F*(1, 21) = 6.34, *p* < 0.05], however the effect of offer failed to reach significance [*F*(1, 21) = 0.245, *p* = 0.626], as well as the interaction [*F*(1, 21) = 0.122, *p* = 0.731]. Importantly, the mentalizing strategy had a significantly stronger effect as compared with distancing for altruistic behavior [*t*(1, 21) = −2.5, *p* < 0.05], but failed to reach significance for selfish behavior [*t*(1, 21) = −1.617, *p* = 0.121]. See Figure 3C.

### Discussion

The aim of this first experiment was twofold. Firstly, we wanted to test whether emotion regulation can be applied in an interpersonal context to complex social emotions, as opposed to the simple visual stimuli used in previous studies. Secondly, we examined whether two different emotion regulation strategies, mentalizing and distancing, can affect emotion perception in an interactive context in which people observed selfish and altruistic behavior regarding the splitting of a pot of money. Our data demonstrate that interpersonal emotion regulation is possible, and indeed strongly affects our perception of both selfish and altruistic behaviors. Importantly, mentalizing (e.g., reinterpretation of the intentions of the players in a way to make them less negative) increased the valence (more positive) of selfish economic offers (in the range of €1–€3 out of 10). Conversely, distancing (e.g., considering events with a detached perspective) did not affect the negative emotions elicited by selfish offers, but paradoxically decreased the valence of emotions elicited by the altruistic offer of €5. Questionnaires confirmed this observation, and suggested that the emotion regulated by the strategies was disappointment (higher values) but also other unpleasant emotions when treated selfishly, and happiness and surprise when treated altruistically. Interestingly, analyses on arousal revealed that mentalizing not only increased the valence of the offers leading recipients to consider them as more positive, but also increased the arousal associated with them (size effect of valence of Figure 2). This result may be in apparent contradiction with a previous experiment (Grecucci et al., 2012), in which authors found that arousal decreased when reappraising IAPS pictures. However, the stimuli used in this other study were very unpleasant, and even when reappraised they remained quite negative images, whereas in the DG subjects changed the valence of selfish proposals, actually considering them as more positive (SAM valence ratings were on the positive range, from 5 to 9 points, except for €1 euro offers).

On the contrary, distancing failed to increase the valence for negative emotions (elicited by selfish proposals), but also decreased the valence of positive emotions elicited by altruistic proposals (offer 5€). In other words, recipients failed to alter the meaning of the proposals. Notably this also affected arousal, but this time decreasing the strength of emotions (size effect of arousal – Figure 2), as they were perceived as still unpleasant (contrary to mentalized trials). Last but not least, the perceived change of emotional strength was stronger when using mentalizing than when using distancing, indicating that mentalizing is a more powerful way to regulate one’s emotions.

## Experiment 2

The aim of Experiment 2 was to test whether emotion regulation is different when applied in social and non-social situations. Participants played the Dictator Game, but with both human (in a similar fashion to Experiment 1) and computer partners. Participants were trained to apply reappraisal when facing human and computer partners. The strategy was the same (cognitive reinterpretation of the event in a way to make it less negative) but with a focus on the intentions in case of a human partner, and a focus on situation when the partner was a computer. We predicted both strategies are effective in altering the emotional experience. However, we expected a stronger effect for interpersonal regulation (greater differences between human and computer in reappraisal condition than in look condition).

### Methods

#### Participants

Twenty-four participants (10 males) from the local population participated in the study, with a mean age of 22.91 years (SD ± 4.77). The local ethics committee approved the study and all participants provided written informed consent after the procedures had been fully explained.

#### Dictator game

The Dictator Game as described above was used, with the only difference that a computer image was presented in the computer condition instead of a face. Participants were told that proposals in the computer condition were randomly generated. Again, each round involved receiving monetary proposals, with each trial dividing 10€. The offers included four repetitions of five possible offers (1€, 2€, 3€, 4€, and 5€ out of 10€), for 20 offers for each of the four conditions (Look vs. Reappraisal, Human vs. Computer), for a total of 80 trials. Type of offers and partners (Computer vs. Human) were completely randomized inside each block, whereas the strategies were separated into two blocks. To encourage engagement in the task it was emphasized that they would be paid a percentage of what they received during the game. Again participants rated their emotions separately on two scales (arousal and valence).

#### Emotion regulation instructions

Before beginning the game, participants were told that they would use a specific cognitive strategy upon receipt of any offer. A written protocol describing reappraisal was provided, very similar to that of Experiment 1, with the exception that the distancing strategy was omitted and also that examples were given as to how to apply reappraisal in both contexts (human vs. computer). To apply reappraisal to a human partner they were asked to focus on the mind of the player, building an interpretation of the intentions behind their behavior. This reinterpretation of their intentions was meant to be less negative. Some examples were then given (“*he is not that stingy, probably does not have so much money to give me*,” “*this is the best he can do*”). To apply reappraisal to a computer partner (non-social regulation) they were asked to focus on the situation, building an interpretation of the event. This reinterpretation was meant to be less negative. Some examples were then given (“*what bad luck*,” “*next time will be better*”). Finally, for the “look” condition, they were to simply allow themselves to respond naturally without any effort of interpretation.

Before beginning the first block of DG, we verified that participants understood the respective emotion regulation instructions by requiring them to verbalize what they would do when confronted with different offers. A practice session proceeded every block.

#### Questionnaires

At the conclusion of the experiment, participants were asked to rate their emotional state when they received the prototypical example of a very unfair (€1 out of €10), and fair offer (€5 out of €10) separately for computer and human partners. Moreover, we asked the strength of perceived emotions when receiving the unfair offer for all conditions (Human vs. Computer, Look vs. Reappraisal). To check for differences on perceived effect of reappraisal between the human and computer partners, at the end of the experiment we asked for ratings on a 9-point Likert scale as to how much they felt their emotion change, for both interacting with a human and with a computer partner. An example of the precise strategies adopted during the experiment was also recorded for every participant (for both strategies) after the experiment.

Additionally, participants completed the Interpersonal Reactivity Index (IRI, Davis, 1980), to test for their ability to take others’ perspective and empathic abilities, and the Emotion Regulation Questionnaire (ERQ, Gross and John, 2003) as a measure of the frequency of reappraisal usage in daily life.

### Results

#### Emotion ratings in the dictator game

We first examined if the affective ratings were different across regulation strategies. We computed two separate ANOVAs, one for valence and one for arousal each with reappraisal Strategies (reappraisal vs. look), Partner (human vs. computer), and Offer type (1€, 2€, 3€, 4€, and 5€) as factors. Analysis on valence returned a significant main effect of Strategy [*F*(1, 23) = 39.724, *p* < 0.0001], of Partner [*F*(4, 84) = 12.363, *p* < 0.005], and of Offers [*F*(4, 92) = 122.299, *p* < 0.0001], as well as a significant Partner × Strategy interaction [*F*(1, 23) = 4.357, *p* < 0.05], Partners × Offers [*F*(4, 92) = 2.792, *p* < 0.05], and Strategy × Offers [*F*(4, 92) = 5.390, *p* < 0.001]. However, the triple interaction was not significant [*F*(4, 92) = 0.101, *p* = 0.982]. Next, we ran Bonferroni-corrected *post hoc* tests with participants’ subjective ratings as dependent variables, comparing between human and computer partner to explore the above 2-way interactions.

**Figure 4:**
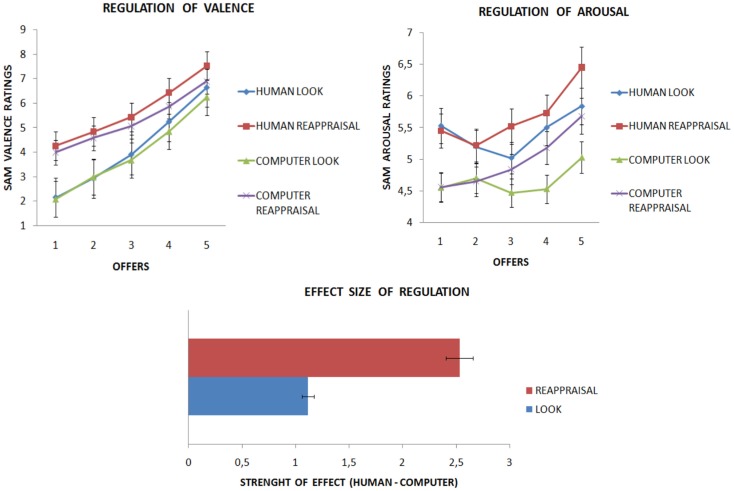
**Results from Experiment 2 are presented**. (Upper part) Subjects were successful in regulating when both interacting with human and computer partners. However, reappraisal was stronger for human than computer partner. Analyses on arousal returned an effect of strategy on offers, according to which only the altruistic offer €5 was perceived in the reappraisal condition stronger than the look condition (independently from partner). (Lower part) Valence change was larger when interacting with human relative to computer partners.

Partner × Strategy contrasts were all significant (computer-look vs. human-look, computer-reappraisal vs. human-reappraisal; computer-look vs. human-reappraisal, computer-reappraisal vs. human-look, *p* < 0.05). Partner × Offer contrast showed significant effects for 4€, and 5€ offers (*p* < 0.05). Strategy × Offer contrasts were all significant (look 1€ vs. reappraising 1€; look 2€ vs. reappraising 2€; look 3€ vs. reappraising 3€; look 4€ vs. reappraising 4€; look 5€ vs. reappraising 5€). These analyses clarified that the strategies affected the valence ratings in different ways when interacting with either a human or a computer partner. Reappraisal had a stronger effect for human partners than for computer partners, whereas in the look condition there was little difference between human partners and computer partners. Moreover, an effect of specific offers made by human and computer partners was visible, being fair offers made by humans perceived as more positive than when made by computers. Last but not least, reappraised offers were rated as more positive than offers simply attended to (see Figure 4), however the strongest effect of reappraisal was found for selfish offers.

**Figure 5:**
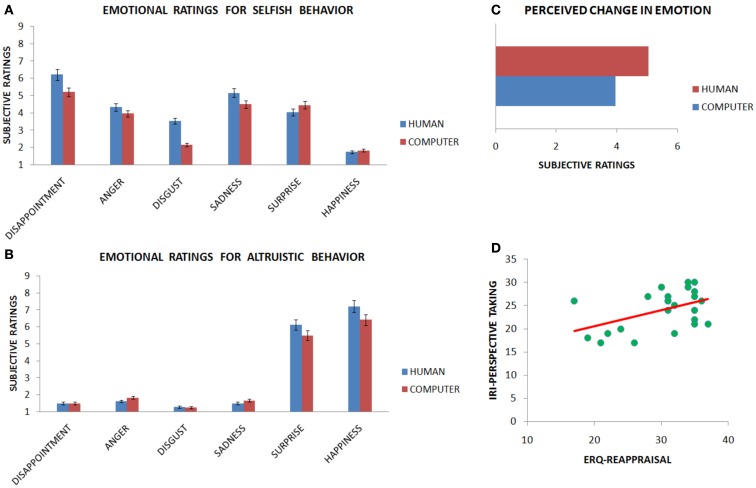
**Questionnaires after Experiment 2 is presented**. Subjects were more disappointed and disgusted when observing a selfish behavior (1€ offer) from human rather than computer partners **(A)**. When receiving altruistic offer (5€ offer) subjects were happier when the donator was a human partner **(B)**. Subjects perceived a change in the strength of emotions when applying the strategies with both partners. However the human condition showed larger effects **(C)**. Lastly, a correlation was observed between the ability to take the perspective of others (IRI questionnaire) and the ability to apply reappraisal **(D)**.

Then, we computed ANOVA on arousal that returned a significant main effect of Partner [*F*(4, 84) = 22.275, *p* < 0.0001], and of Offer [*F*(4, 92) = 4.502, *p* < 0.005], but not of Strategy [*F*(1, 23) = 2.714, *p* = 0.113]. Moreover, there was an interaction between Strategy × Offer [*F*(4, 92) = 3.617, *p* < 0.01], but not of Partner × Strategy [*F*(1, 23) = 0.141, *p* = 0.711], nor Partner × Offer [*F*(4, 92) = 1.835, *p* = 0.129]. The same applied for the triple interaction [*F*(4, 92) = 0.741, *p* = 0.566]. To explore the Strategy × Offer interaction, we ran Bonferroni-corrected *post hoc* tests on arousal ratings between strategies, for every offer. These returned a significant difference for the most fair offer (€5: *p* < 0.05). In other words, arousal was stronger when the €5 offer was reappraised rather than when it was simply attended to. See Figure 5.

To test the hypothesis of a stronger effect of reappraisal in changing the perceived valence for human as compared to computer offers, we computed the effect size of valence change separately for each condition. This measure was calculated as the difference between perceived valence when attending a human vs. a computer on one hand, and when reappraising a human vs. a computer on the other. We predict larger differences when applying the reappraisal strategy than when simply looking at different partners. While the difference in the look condition between playing with a computer compared with a human was 1.11 points, the difference between partners in the reappraisal condition was of 2.53 points, meaning that reappraisal doubled the difference between playing with a human or with a computer (see Figure 4 and Table 2).

**Table 2:** **Experiment 2-results from the experiment and from the questionnaires**.

Experiment ratings				

	Valence ratings			
	Computer-look	Computer-reappraisal	Human-look^^^	Human-reappraisal^^^
€1*	2.11 (1.17)	4.06 (1.41)	2.36 (1.18)	4.55 (1.50)
€2*	3.02 (1.29)	4,77 (1.53)	3.06 (1.35)	5.01 (1.25)
€3*	3.67 (1.19)	5.05 (1.17)	3.94 (1.24)	5.48 (1.48)
€4*§	4.86 (1.11)	5.77 (1.26)	5.25 (1.22)	6.44 (1.15)
€5*§	6.26 (1.30)	6.71 (1.51)	6.56 (1.16)	7.39 (1.37)

	**Arousal ratings**			
	**Computer-look**	**Computer-reappraisal**	**Human-look**	**Human-reappraisal**

€1	4.60 (2.11)	4.65 (1.79)	5.43 (2.16)	5.76 (1.74)
€2	4.76 (1.69)	4.69 (1.53)	5.07 (1.72)	5.43 (1.80)
€3	4.53 (1.46)	4.94 (1.58)	5.07 (1.68)	5.72 (1.92)
€4	4.55 (1.57)	5.06 (1.77)	5.55 (1.48)	5.69 (1.91)
€5§	4.97 (1.75)	5.43 (2.01)	5.81 (1.66)	6.14 (2.20)

**Questionnaires**

	**Offer 1€**		**Offer 5€**	
	**Human**	**Computer**	**Human**	**Computer**

Disappointment	6.20 (2.22)**	5.20 (2.37)	1.5 (0.78)	1.5 (1.02)
Anger	4.33 (2.07)	3.95 (2.29)	1.62 (1.34)	1.83 (1.40)
Disgust	3.54 (2.26)**	2.16 (1.85)	1.29 (0.85)	1.25 (0.67)
Sadness	5.16 (1.97)	4.5 (2.53)	1.5 (0.83)	1.66 (0.81)
Surprise	4.04 (2.42)	4.45 (2.35)	6.12 (2.13)	5.5 (2.39)
Happiness	1.75 (1.18)	1.83 (1.01)	7.20 (1.35)**	6.41 (2.18)

*Indicates a significant difference at the level of Strategy × Offer interaction.

*^^^Indicates a significant difference at the level of Partner × Strategy interaction*.

*§Indicates a significant difference at the level of Partner × Offer interaction*.

**Indicates a significant difference between partners inside every offer.

#### Questionnaires

Subjective ratings when receiving the most fair (€5) and unfair (€1) offers were entered into an ANOVA for each of the six queried emotions (anger, disgust, surprise, sadness, happiness, and disappointment) for both human and computer partners. Analyses returned a significant main effect of Partner [*F*(1, 23) = 4.385, *p* < 0.05], of Offer [*F*(1, 23) = 16.314, *p* < 0.001], and of Emotion [*F*(1, 23) = 24.356, *p* < 0.0001], as well as a significant Offer × Emotion interaction [*F*(5, 115) = 101.034, *p* < 0.0001], and the triple interaction [*F*(5, 115) = 3.856, *p* < 0.005].

Next, we ran Fisher-corrected *post hoc* tests with participants’ subjective ratings as dependent variables to compare between human and computer partners for each emotion and every offer. For the selfish unfair offers, disgust, and disappointment elicited when playing with a human were stronger than when playing with a computer partner (*p* < 0.05, respectively: score = 3.54 vs. 2.16, score = 6.2 vs. 5.2). The other emotions were not statistically significant (all *p* > 0.05).

For the altruistic fair offers, only happiness was stronger for human than computer partners (*p* < 0.05, score = 7.21 vs. 6.41). The other emotions were not statistically significant (all *p* > 0.05). See Figures 5A,B and Table 2. Participants felt their emotions change more strongly when interacting with a human rather a computer partner [respectively, 5.04 and 3.95, *t*(1, 23) = −3.137, *p* < 0.005]. See Figure 5C.

Analysis of questionnaires revealed a positive correlation between the reported frequency of reappraisal usage in daily life (ERQ-reappraisal subscale) and the ability to take the psychological point of view of others (IRI-perspective taking subscale; rho = 0.471, *p* < 0.01). See Figure 5D.

### Discussion

The aim of this study was to test for differences in the regulation of emotions stemming from interaction with human and non-human partners respectively. Results indicated that even though reappraisal can be successfully applied to both contexts, participants showed a stronger effect on their perceived valence when playing with a human partner. Therefore, it seems that reappraisal leads participants to change the valence of their emotions to make them more positive for selfish offers, but also stronger and more vivid for fair offers. Moreover, emotional ratings indicated that on one hand, participants were more disappointed and disgusted when recipients of selfish behavior from human rather than computer partners, however when receiving altruistic offers participants were happier when the Allocator was a human partner.

Last but not least, there was a positive correlation between IRI and ERQ questionnaires, indicating that the ability to take the psychological point of view of others and emotion regulation abilities are related. Indeed, the IRI (perspective taking subscale) addresses one’s tendency to take another’s point of view, akin to “theory of mind” (Davis, 1983; Frías-Navarro, 2009). This ability is essential when reappraising the intentions of the other players (mentalizing).

## General Discussion

Our ability to regulate emotions when interacting with others is considered to be a crucial dimension of both emotional intelligence (Mayer and Salovey, 1997; Lopes et al., 2011), and of good mental health (Gross, 2002; van’tWout et al., 2010). Despite the extensive literature on emotion “self regulation” (see Ochsner and Gross, 2008), evidence of emotion regulation in social interactive situations is still poorly understood. In the present study, we examined whether emotion regulation strategies can be successfully applied to socially driven emotions. This is especially important when considering that emotion regulation typically occurs in social contexts (Rottenberg et al., 2005). Previous research has demonstrated that emotion regulation strategies based on the reinterpretation of an event as less negative are powerful tools to allow us to reduce the subjective experience toward emotional unpleasant pictures. However, few attempts have been made to extend these findings to the domain of interpersonal emotions. To elicit these kind of emotions we exposed participants to altruistic and selfish behaviors while playing the Dictator Game as Recipients (Kahneman et al., 1986). Prior to both experiments, participants were trained to apply different forms of reappraisal strategies (mentalizing vs. distancing in Experiment 1), and toward human and non-human partners (Experiment 2).

Firstly, our data demonstrate that emotion regulation can be successfully applied to socially driven emotions. Across both experiments participants reported an increase in valence (that is, less unpleasant emotions) when reappraising the intentions behind both selfish and altruistic behavior. More importantly, Experiment 1 showed that not all emotion regulation strategies are equally good at altering our emotional responses. While mentalizing-based reappraisal (defined as the “*reinterpretation of the intentions of the player in a way to make them less negative*,” Grecucci et al., 2012) was effective in increasing the valence of the emotions experienced, distancing-based reappraisal (“*putting oneself in a detached perspective*”) was not. Paradoxically, avoiding emotions (as a consequence of a distancing strategy), not only failed to decrease the unpleasantness of experienced emotions when treated selfishly, but interestingly also decreased the pleasantness of emotions elicited by altruistic behaviors. Because psychiatric disorders are largely characterized by excessive negative emotions (Werner and Gross, 2010), this strategy may therefore lead to emotional disturbance rather than emotional relief.

Experiment 2 tested whether reappraisal can also be used when the emotion elicited comes from a non-human partner. This is important to appreciate differences in emotional regulation when applied to social and non-social contexts. Even though both conditions showed a modulation of emotional valence when receiving selfish proposals, there was a difference of partner type. Valence change was stronger when participants regulated their emotions in response to human offers. In fact, when comparing human and computer in the baseline condition, this difference was doubled in the reappraisal condition. Arousal analyses showed interesting differences in increasing the strength of vividness of experienced emotions when they were associated with an altruistic behavior.

Both experiments showed interesting results regarding the perception of the strength of the emotional experience, i.e., arousal. When using reappraisal based on cognitive reinterpretation, both experiments showed that once unpleasant (and at a lesser extent also positive) emotions are changed in terms of their valence (perceived as less unpleasant) arousal is increased (evident for €5 offer in experiment 2), meaning that emotion regulation strategies that are effective in reframing the events in a more positive way let us experience our emotions more vividly. In contrast, Experiment 1 showed that distancing-based reappraisal did not change the experienced emotion (unpleasant emotions in response to selfish offers are still perceived as unpleasant, and pleasant emotions in response to fair offers are even less pleasant). One conclusion is therefore that not all strategies are effective to the same extent in regulating our emotions. Even though distancing may mitigate individuals’ experience of their emotions by avoiding them, in the long run it can lead individuals to progressively detach from others and from situations. This in turn may lead to anhedonia and isolation as shown by many psychiatric disorders (Leising et al., 2006; Ballon et al., 2007; Gunderson, 2007). By definition, emotion regulation is maladaptive “*when it does not change the emotional response in the desired way (e.g., decrease negative affect) or when the long term costs (decreased work, social functioning, vitality) outweigh the benefits of short-term changes in emotion (relief, temporary decrease in anxiety)*” (cfr. Werner and Gross, 2010). From our results, distancing may have a temporary relieving effect by decreasing arousal, but at the cost of not changing or even increasing their unpleasantness.

Psychological studies have shown that cognitive reappraisal is one of the most flexible and adaptive strategies for regulating negative emotions (Gross, 2002). The present study confirms previous findings, but also extends these results into the domain of interpersonal emotion regulation. In particular, Grecucci et al. (2012) proposed a variation of reappraisal, called mentalizing-reappraisal that merges previous work on the importance of building a mental representation of others’ minds (Frith and Frith, 2003), and its effect on the regulation of the interpreter’s emotional state (Fonagy, 2006). In practical situations, mentalizing strategies are commonly implemented in psychological treatment of anxiety disorders, borderline personality disorders, eating disorders, and childhood problems (Clarkin et al., 2006; Fonagy, 2006; Bateman and Fonagy, 2011; Lemma et al., 2011).

The present experiment also extends previous findings on decision-making. Broadly speaking, emotion regulation strategies applied to decision-making have one notable advantage as compared to basic emotion regulation studies: they have the opportunity to study complex emotions that cannot be elicited in simple visual stimuli tasks. Emotions elicited by the outcome of our decisions are of a qualitatively different nature than those experienced while simply watching disturbing images, and so it was an open question whether these strategies can be effective in regulating such emotions and influencing decision behavior in real-life. In everyday life we are typically confronted with a variety of emotions directly induced by decisions, by the evaluation of risks and possible losses, and last but not least by social interactions, and emotion regulation seems particularly useful in such contexts. Therefore, investigating whether emotion regulation strategies can have an effect in decision-making contexts has the opportunity to extend emotion regulation research beyond affective responses to simple emotional pictures into more complex scenarios. Social norms, such as fairness, equality, and cooperation, play a fundamental role in societies (Deutsch, 1975; Coleman, 1990), with these norms influencing not only our decisions when balancing self-interest with others’ interest, but also our perception of the decisions of others that affect us. Indeed, people tend to select the most cooperative individuals, and those who contribute less than others are generally left out of social exchanges (Barclay, 2004; Coricelli et al., 2004; Barclay and Willer, 2007; Cornelissen et al., 2011). Using the Recipient role of the Dictator Game permits exploration of how we react to social norm violations. In both experiments we were able to show that when receiving selfish offers participants reacted to them with unpleasant emotions, linearly increasing with the unfairness of the monetary offer. The detection of violations from social norms (Montague and Lohrenz, 2007) may be of great importance for future interactions with and eventual punishment of self-interested individuals. This is shown by the comparison between human and computer partners’ offers, where fair offers are perceived as more pleasant when the partner was a human, whereas unfair offers elicited more negative emotions.

On the same line, when reappraising, the identity of the player matters: we are more prone to “excuse” the selfishness of a human rather than a non-human donor. The justification of occasional violations of social norms may be functional in keeping cooperation high between individuals belonging to the same group.

In recent years, progress in understanding the neural mechanisms of emotional regulation has used functional imaging to identify the neural signatures of regulation (Ochsner and Gross, 2005). The neural bases of different strategies have been outlined, as well as how these processes act on target regions responsible for the specific emotion involved. For example, imaging studies have shown that reappraisal activates systems appearing to modulate activity in neural systems associated with emotional responding, such as the amygdala (Beauregard et al., 2001; Ochsner et al., 2002, 2004; Levesque et al., 2003; Kalisch et al., 2005; Ochsner and Gross, 2005; Phan et al., 2005; Urry et al., 2006; Banks et al., 2007; Kim and Hamann, 2007). However, the role of emotion regulation for socially driven emotions remains quite poorly explored. Just two studies have explored the neural mechanisms behind social emotion regulation (Koenigsberg et al., 2011; Grecucci et al., 2012). However, these studies used pictures with social content but not emotions stemming from real social situations (Koenigsberg et al., 2011), or the effect of emotion regulation was observed indirectly (Grecucci et al., 2012). Future experiments based on the paradigm developed in this study can be fruitfully transferred to neuroimaging experiments to uncover the brain bases of the regulation of socially driven emotions, or more importantly try to use physiological measure of emotion regulation such as galvanic skin responses to test for implicit indexes of emotion regulation abilities. These implicit measures do not suffer from the expectations participants develop following the instructions and thus can be more reliable then subjective ratings.

In conclusion, we investigated the effect of reappraisal based emotion regulation strategies, and further looked at the effects of playing with a human or a non-human (computer) partner. We believe these results are important as they shed light on two points: the possibility of regulating socially driven emotions on one hand, and the effect of different strategies themselves on the other. Our results show that emotional reappraisal specifically influences emotions stemming from the interaction with altruistic and selfish proposers. Both emotions elicited by altruistic and selfish offers showed an effect of regulation for the two main dimensions of emotional experience: valence and arousal. These results extend previous findings on this topic and hold the promise of shedding light on the understanding of interpersonal problems shown by psychiatric populations due to poor emotion regulation (Werner and Gross, 2010).

## Conflict of Interest Statement

The authors declare that the research was conducted in the absence of any commercial or financial relationships that could be construed as a potential conflict of interest.

## Appendix

### Simplified version of the emotion regulation strategies

#### Mentalizing strategy

The way we perceive an event may alter the perception of the event in a way to make it more or less negative.

See for example the following image:

One interpretation can be that this woman is suffering because of the death of a beloved one. Another interpretation is that she is simply tired. Both are plausible interpretations, but the effect of these instructions may be different. The first increases the perceived negativity of the event, the second decreases it. We are asking you to make an effort of reinterpretation of the event in a way to decrease its negativity…

Can you generate another example of how to reinterpret that picture as less negative?…

Now we will teach you how to apply this strategy to the domain of the Dictator Game.

In the following part of the experiment you are asked to reinterpret the intentions of your partner in a way to consider them as less negative…

Subjects were given some examples on how to apply this strategy to DG:

“You can think that this person has no money to give you,” “He/she is in troubles,” “In another situation he/she may be more generous”

#### Distancing strategy

Another useful strategy that people can use to decrease the negativity of an event, is to take the distance from it.

See for example the following image:

Such a situation is undoubtedly unpleasant. However, the fact that we are more or less involved in this situation determines how negative we perceive that situation. Someone can think that this situation has great relevance for himself/herself and perceive it as very negative. Someone else may in turn think it does not affect his/her life.

These two ways of thinking, in touch or detached from the situation, alter the way we perceive that situation…

Can you generate another example of how to think in a detached way that picture?…

Now we will teach you how to apply this strategy to the domain of the Dictator Game.

When asked to apply such a strategy you should put yourself in a detached perspective and think that this situation is not relevant for you.

Subjects were then given some examples on how to apply this strategy to DG:

“This offer won’t affect your economic situation,” “I don’t care of your money,” “I don’t even know you”

#### Baseline “look” condition

Look at the offers and make your response in a spontaneous way without applying any strategy.

## References

[B1] Ballon J. S. Kaur T. Marks I. I. Cadenhead K. S. (2007). Social functioning in young people at risk for schizophrenia. Psychiatry Res. 151, 29–35 10.1016/j.psychres.2006.10.012 17383739 PMC3065359

[B2] Banks S. J. Eddy K. T. Angstadt M. Nathan P. J. Phan K. L. (2007). Amygdala frontal connectivity during emotion regulation. Soc. Cogn. Affect. Neurosci. 2, 303–312 10.1093/scan/nsm029 18985136 PMC2566753

[B3] Barclay P. (2004). Trustworthiness and competitive altruism can also solve the “tragedy of the commons.” Evol. Hum. Behav. 25, 209–220 10.1016/j.evolhumbehav.2004.04.002

[B4] Barclay P. Willer R. (2007). Partner choice creates competitive altruism in humans. Proc. Biol. Sci. 274, 749–753 10.1098/rspb.2006.0209 17255001 PMC2197220

[B5] Bateman A. W. Fonagy P. (2011). “Borderline personality disorder,” in Handbook of Mentalizing in Mental Health Practice, eds Bateman A. W. Fonagy P. (Arlington: Amer Psychiatric Pub), 273–288

[B6] Beauregard M. Levesque J. Bourgouin P. (2001). Neural correlates of conscious self-regulation of emotion. J. Neurosci. 21, RC165 11549754 10.1523/JNEUROSCI.21-18-j0001.2001 PMC6763007

[B7] Clarkin J. F. Yeomans F. E. Kernberg O. F. (2006). Psychotherapy for Borderline Personality. Focusing on Object Relations. Washington, DC: American Psychiatric Press

[B8] Coleman J. S. (1990). Foundations of Social Theory. Cambridge, MA: Harvard University Press

[B9] Coricelli G. Fehr E. Fellner G. (2004). Partner selection in public goods experiments. J. Conflict Resolut. 48, 356–378 10.1177/0022002704264143

[B10] Cornelissen G. Dewitte S. Warlop L. (2011). Are social value orientations expressed automatically? Decision making in the dictator game. Pers. Soc. Psychol. Bull. 37, 1–11 10.1177/0146167211405996 21518808

[B11] Davis M. H. (1980). A multidimensional approach to individual differences in empathy. JSAS Catalog Sel. Doc. Psychol. 10, 85

[B12] Davis M. H. (1983). Measuring individual differences in empathy: evidence for a multidimensional approach. J. Pers. Soc. Psychol. 44, 113–126 10.1037/0022-3514.44.1.113

[B13] Deutsch M. (1975). Equity, equality, and need: what determines which value will be used as the basis of distributive justice? J. Soc. Issues 31, 131–149 10.1111/j.1540-4560.1975.tb01000.x

[B14] Eippert F. Veit R. Weiskopf N. Erb M. Birbaumer N. Anders S. (2007). Regulation of emotional responses elicited by threat-related stimuli. Hum. Brain Mapp. 28, 409–423 10.1002/hbm.20291 17133391 PMC6871321

[B15] Evans S. Fleming S. M. Dolan R. J. Averbeck B. B. (2011). Effects of emotional preferences on value-based decision-making are mediated by mentalizing not reward networks. J. Cogn. Neurosci. 23, 2197–2210 10.1162/jocn.2010.21584 20946058 PMC3258492

[B16] Fehr E. Gächter S. (2002). Altruistic punishment in humans. Nature 415, 137–140 10.1038/415269a 11805825

[B17] Fonagy P. (2006). “The mentalization-focused approach to social development,” in Handbook of Mentalization-Based Treatment, eds Allen J. G. Fonagy P. (Chichester: John Wiley & Sons Ltd), 53–100

[B18] Frías-Navarro D. (2009). Davis’ Interpersonal Reactivity Index (IRI). Valencia: Valencia Universidad de Valencia [Manuscript not published].

[B19] Frith U. Frith C. D. (2003). Development and neurophysiology of mentalizing. Philos. Trans. R. Soc. Lond. B Biol. Sci. 358, 459–473 10.1098/rstb.2002.1218 12689373 PMC1693139

[B20] Frith U. Morton J. Leslie A. M. (1991). The cognitive basis of a biological disorder-autism. Trends Neurosci. 14, 433–438 10.1016/0166-2236(91)90041-R 1722361

[B21] Grecucci A. Giorgetta C. Van’tWout M. Bonini N. Sanfey A. G. (2012). Reappraising the ultimatum: an fMRI study of emotion regulation and decision making. Cereb. Cortex. [Epub ahead of print]. 22368088 10.1093/cercor/bhs028

[B22] Gross J. J. (1998). The emerging field of emotion regulation: an integrative review. Rev. Gen. Psychol. 2, 271–299 10.1037/1089-2680.2.3.271

[B23] Gross J. J. (1999). Emotion regulation: past, present, future. Cogn. Emot. 13, 551–573 10.1080/026999399379186

[B24] Gross J. J. (2002). Emotion regulation: affective, cognitive, and social consequences. Psychophysiology 39, 281–291 10.1017/S0048577201393198 12212647

[B25] Gross J. J. John O. P. (2003). Individual differences in two emotion regulation processes: implications for affect, relationships, and well-being. J. Pers. Soc. Psychol. 85, 348–362 10.1037/0022-3514.85.2.348 12916575

[B26] Gunderson J. G. (2007). Disturbed relationships as a phenotype for borderline personality disorder. Am. J. Psychiatry 164, 1637–1640 10.1176/appi.ajp.2007.07050727 17974925

[B27] Guth W. Schmittberger R. Schwarz B. (1982). An experimental analysis of ultimatum bargaining. J. Econ. Behav. Organ. 3, 376–388 10.1016/0167-2681(82)90011-7

[B28] Kahneman D. Knetsch J. L. Thaler R. H. (1986). Fairness and the assumptions of economics. J. Business 59, 285–300 10.1086/296367

[B29] Kalisch R. Wiech K. Critchley H. D. Seymour B. O’Doherty J. P. Oakley D. A. (2005). Anxiety reduction through detachment: subjective, physiological, and neural effects. J. Cogn. Neurosci. 17, 874–883 10.1162/0898929054021184 15969906

[B30] Kim S. H. Hamann S. (2007). Neural correlates of positive and negative emotion regulation. J. Cogn. Neurosci. 19, 776–798 10.1162/jocn.2007.19.5.776 17488204

[B31] Koenigsberg H. W. Fan J. Ochsner K. N. Liu X. Guise K. Pizzarello S. (2011). Neural correlates of using distancing to regulate emotional responses to social situations. Neuropsychologia 48, 1813–1822 10.1016/j.neuropsychologia.2010.03.002 20226799 PMC2905649

[B32] Lang P. J. (1994). Measuring emotion: the self-assessment manikin and the semantic differential. J. Behav. Ther. Exp. Psychiatry 25, 49–59 10.1016/0005-7916(94)90063-9 7962581

[B33] Lang P. J. Bradley M. M. (2010). Emotion and the motivational brain. Biol. Psychol. 84, 437–450 10.1016/j.biopsycho.2009.10.007 19879918 PMC3612949

[B34] Leising D. Sporberg D. Rehbein D. (2006). Characteristic interpersonal behavior in dependent and avoidant personality disorder can be observed within very short interaction sequences. J. Pers. Disord. 20, 319–330 10.1521/pedi.2006.20.4.319 16901257

[B35] Lemma A. Target M. Fonagy P. (2011). Brief Dynamic Interpersonal Therapy: A Clinician’s Guide. Oxford University Press

[B36] Levesque J. Eugene F. Joanette Y. Paquette V. Mensour B. Beaudoin G. (2003). Neural circuitry underlying voluntary suppression of sadness. Biol. Psychiatry 53, 502–510 10.1016/S0006-3223(02)01817-6 12644355

[B37] Lopes P. N. Nezlek J. B. Extremera N. Hertel J. Fernandez-Berrocal P. Schutz A. (2011). Emotion regulation and the quality of social interaction: does the ability to evaluate emotional situations and identify effective responses matter? J. Pers. 79, 429–467 10.1111/j.1467-6494.2010.00689.x 21395594

[B38] Mayer J. D. Salovey P. (1997). “What is emotional intelligence?” in Emotional Development and Emotional Intelligence: Educational Implications, eds Salovey P. Sluyter D. J. (New York: Basic Books), 3–31

[B39] Montague P. R. Lohrenz T. (2007). To detect and correct: norm violations and their enforcement. Neuron 56, 14–18 10.1016/j.neuron.2007.09.020 17920011

[B40] Myerson R. B. (1997). Game Theory: Analysis of Conflict. P. 1. Harvard University Press

[B41] Ochsner K. N. Bunge S. A. Gross J. J. Gabrieli J. D. (2002). Rethinking feelings: an fMRI study of the cognitive regulation of emotion. J. Cogn. Neurosci. 14, 1215–1229 10.1162/089892902760807212 12495527

[B42] Ochsner K. N. Gross J. J. (2005). The cognitive control of emotion. Trends Cogn. Sci. (Regul. Ed.) 9, 242–249 10.1016/j.tics.2005.06.004 15866151

[B43] Ochsner K. N. Gross J. J. (2008). Cognitive emotion regulation: insights from social cognitive and affective neuroscience. Curr. Dir. Psychol. Sci. 17, 153–158 10.1111/j.1467-8721.2008.00566.x PMC4241349 25425765

[B44] Ochsner K. N. Ray R. D. Cooper J. C. Robertson E. R. Chopra S. Gabrieli J. D. (2004). For better or for worse: neural systems supporting the cognitive down- and up-regulation of negative emotion. Neuroimage 23, 483–499 10.1016/j.neuroimage.2004.06.030 15488398

[B45] Phan K. L. Fitzgerald D. A. Nathan P. J. Moore G. J. Uhde T. W. Tancer M. E. (2005). Neural substrates for voluntary suppression of negative affect: a functional magnetic resonance imaging study. Biol. Psychiatry 57, 210–219 10.1016/j.biopsych.2004.10.030 15691521

[B46] Phillips M. L. Drevets W. C. Rauch S. L. Lane R. (2003). Neurobiology of emotion perception II: implications for major psychiatric disorders. Biol. Psychiatry 54, 515–528 10.1016/S0006-3223(03)00168-9 12946880

[B47] Rottenberg J. Gross J. J. Gotlib I. H. (2005). Emotion context insensitivity in major depressive disorder. J. Abnorm. Psychol. 114, 627–639 10.1037/0021-843X.114.4.627 16351385

[B48] Sanfey A. G. Dorris M. (2009). “Games in humans and non-human primates: scanners to single unit,” in Neuroeconomics, eds Glimcher P. W. Camerer C. F. Fehr E. Poldrack R. A. (London: Elsevier), 63–80

[B49] Sanfey A. G. Rilling J. K. Aronson J. A. Nystrom L. E. Cohen J. D. (2003). The neural basis of economic decision-making in the ultimatum game. Science 300, 1755–1758 10.1126/science.1082976 12805551

[B50] Sharp C. Pane H. Ha C. Venta A. Patel A. B. Sturek J. (2011). Theory of mind and emotion regulation difficulties in adolescents with borderline traits. J. Am. Acad. Child Adolesc. Psychiatry 50, 563–573 10.1016/j.jaac.2011.01.017 21621140

[B51] Urry H. L. van Reekum C. M. Johnstone T. Kalin N. H. Thurow M. E. Schaefer H. S. (2006). Amygdala and ventromedial prefrontal cortex areinversely coupled during regulation of negative affect and predict the diurnalpattern of cortisol secretion among older adults. J. Neurosci. 26, 4415–4425 10.1523/JNEUROSCI.3215-05.2006 16624961 PMC6673990

[B52] van’tWout M. Chang L. J. Sanfey A. G. (2010). The influence of emotion regulation on social interactive decision-making. Emotion 10, 815–821 10.1037/a0020069 21171756 PMC3057682

[B53] Vrtickaa P. Sandera D. Vuilleumiera P. (2011). Effects of emotion regulation strategy on brain responses to the valence and social content of visual scenes. Neuropsychologia 49, 1067–1082 10.1016/j.neuropsychologia.2011.02.020 21345342

[B54] Werner K. Gross J. J. (2010). “Emotion regulation and psychopathology. A conceptual framework,” in Emotion Regulation and Psychopathology: A Transdiagnostic Approach to Etiology and Treatment, eds Kring A. M. Sloan D. M. (New York: The Guilford Press), 13–37

